# Braun Lipoprotein Protects against Escherichia coli-Induced Inflammatory Responses and Lethality in Mice

**DOI:** 10.1128/spectrum.03541-22

**Published:** 2023-03-14

**Authors:** Yuan Shen, Zhiguo Gong, Shuangyi Zhang, Jinshan Cao, Wei Mao, Yunhe Fu, Niri Su, Yulin Ding, Jiamin Zhao, Baichen Gu, Shuang Feng, Bo Liu

**Affiliations:** a Laboratory of Veterinary Clinical Pharmacology, College of Veterinary Medicine, Inner Mongolia Agricultural University, Hohhot, China; b Laboratory of Veterinary Public Health, College of Veterinary Medicine, Inner Mongolia Agricultural University, Hohhot, China; c Key Laboratory of Clinical Diagnosis and Treatment Techniques for Animal Disease, Ministry of Agriculture, Inner Mongolia Agricultural University, Hohhot, China; d School of Public Health, Inner Mongolia Medical University, Hohhot, China; e Department of Clinical Veterinary Medicine, College of Veterinary Medicine, Jilin University, Changchun, China; Institute of Microbiology Chinese Academy of Sciences

**Keywords:** Braun lipoprotein, protective roles, *Escherichia coli*, inflammatory responses, lethality

## Abstract

Escherichia coli (E. coli), a Gram-negative bacterium, is an important pathogen that causes several mammalian diseases. The outer membrane components of E. coli, namely, lipopolysaccharide (LPS) and bacterial lipoprotein, can induce the host innate immune response through pattern recognition receptors (PRRs). However, the detailed roles of the E. coli Braun lipoprotein (BLP) in the regulation of host inflammatory response to E. coli infection remain unclear. In this study, we sought to determine the effects of BLP on E. coli-induced host inflammatory response and lethality using mouse models. Experiments using the E. coli DH5α strain (BLP-positive), E. coli JE5505 strain (BLP-negative), and E. coli JE5505 strain combined with BLP indicated that the presence of BLP could alleviate mortality and organ (liver and lung) damage and decrease proinflammatory cytokine (tumor necrosis factor alpha [TNF-α] and interleukin-1β [IL-1β]) and chemokine (regulated on activation normal T-cell expressed and secreted [RANTES]) production in mouse serum and organs. Conversely, E. coli JE5505, E. coli DH5α strain, and E. coli JE5505 combined with BLP treatment induce enhanced anti-inflammatory cytokine (interleukin 10 [IL-10]) production in mouse serum and organs. In addition, BLP could regulate the secretion of proinflammatory cytokines (TNF-α and IL-1β), chemokines (RANTES), and anti-inflammatory factors (IL-10) through mitogen-activated protein kinase (MAPK) and nuclear factor-kappaB (NF-κB) signaling pathways in macrophages. Altogether, our results demonstrate that the bacterial component BLP plays crucial and protective roles in E. coli-infected mice, which may influence the outcome of inflammation in host response to E. coli infection.

**IMPORTANCE** In this study, we investigated the roles of bacterial outer membrane component BLP in regulating inflammatory responses and lethality in mice that were induced by a ubiquitous and serious pathogen, Escherichia coli. BLP could alleviate the mortality of mice and organ damage, as well as decrease proinflammatory cytokines and chemokine production and enhance anti-inflammatory cytokine production in mouse serum and organs. Overall, our results demonstrate that the bacterial component BLP plays crucial and protective roles in E. coli-infected mice through regulating the production of an inflammatory mediator, which may influence the outcome of inflammation in host response to E. coli infection. Our findings provide new information about the basic biology involved in immune responses to E. coli and host-bacterial interactions, which have the potential to translate into novel approaches for the diagnosis and treatment of E. coli-related medical conditions, such as bacteremia and sepsis.

## INTRODUCTION

Escherichia coli (E. coli) is a major commensal Gram-negative bacterium (1). Some E. coli strains can cause diverse diseases, such as urinary tract infection, bloodstream infection, and sepsis (2). Pathogenic E. coli strains can be divided into two categories, as follows: one category causes intestinal pathologies while the other category causes extraintestinal pathologies (3). A strong relationship exists between inflammatory response and organ damage during the E. coli infection process in hosts (4). This process begins when pathogen-associated molecular patterns (PAMPs) are recognized by pattern recognition receptors (PRRs) in the host innate immune system, which results in the phosphorylation of several proteins following the expression of inflammatory mediators, including cytokines and chemokines (5). Hence, the innate immune system is crucial for host defenses against E. coli infection.

lipopolysaccharide (LPS), a prototype PAMP from the cell walls of Gram-negative bacteria, such as E. coli, is recognized by Toll-like receptor 4 (TLR4) in complex with CD14 and MD2 and thus activates TLR4-mediated signaling cascades. As a result, the activation of the transcription factor nuclear factor-kappaB (NF-κB) and the mitogen-activated protein kinase (MAPK) signaling pathway and the secretion of proinflammatory cytokines and chemokines, which mobilize and activate immune cells, are triggered (6–9). In addition to LPS, bacterial lipoproteins have been found in the cell envelope of E. coli and studied for their chemical characterization, spatial distribution, and function. Braun lipoprotein (BLP) from E. coli is one of the most abundant components of the outer membrane, characterized by its modified N-terminal cysteine residue, which contains three palmityl residues, and is structured as a triacylcysteinyl-modified peptide (10). BLP exists in the following two states: ~33% remains covalently bound to the peptidoglycan on the cell wall through peptide bonds and ~66% remains free in the periplasm (11). BLP is a Toll-like receptor 2 (TLR2) agonist that is more potent than its synthetic structural analogue Pam3CysSK4 (12). BLP is an immunobiologically active compound of E. coli membranes that induces an LPS-like endotoxic response in the host through TLR2 activation (13). Accordingly, the endotoxin activity of E. coli is not only due to LPS but also dependent on the presence of BLP. Bacterial lipoproteins activate monocytes and macrophages to produce inflammatory cytokines and induce lethal shock in both LPS-reactive C3H/HeN mice and LPS-hyporesponsive C3H/HeJ mice (14–16).

Tolerance is a vital aspect of the host’s defense against infection, which could decrease the host’s tissue damage caused by the pathogens or the immune response against pathogens. (17, 18). Previous studies have shown that low-dose prestimulation of LPS in monocytes or macrophages results in a hyporeactive state that reduces proinflammatory cytokine production and protects mice against lethality induced by subsequent exposure to high-dose LPS (19, 20). In addition, tolerance induced by synthetic bacterial lipoproteins has been demonstrated to prevent mortality induced by bacterial lipoproteins, LPS, live bacteria, and polybacterial sepsis (21). This protective regulatory mechanism, developed during evolution, provides resistance to subsequent exposure to lethal doses of bacterial lipoproteins, Staphylococcus aureus, Salmonella enterica serovar Typhimurium infection, and bacterial sepsis caused by cecal ligation and puncture (21, 22). These findings highlight the importance of bacterial lipoproteins in regulating the host immune response to E. coli infection. However, the exact roles of BLP in modulating the host inflammatory response in animal models remains unclear.

Here, we hypothesized that the BLP of E. coli represents an immunobiologically active compound with roles in regulating the outcome of E. coli-induced host inflammatory response. Thus, in the present study, the effects of the E. coli outer membrane component BLP on E. coli-induced host inflammatory response and lethality were examined in mouse models.

## RESULTS

### E. coli JE5505 has enhanced lethality compared with E. coli DH5α in mice.

**FIG 1:**
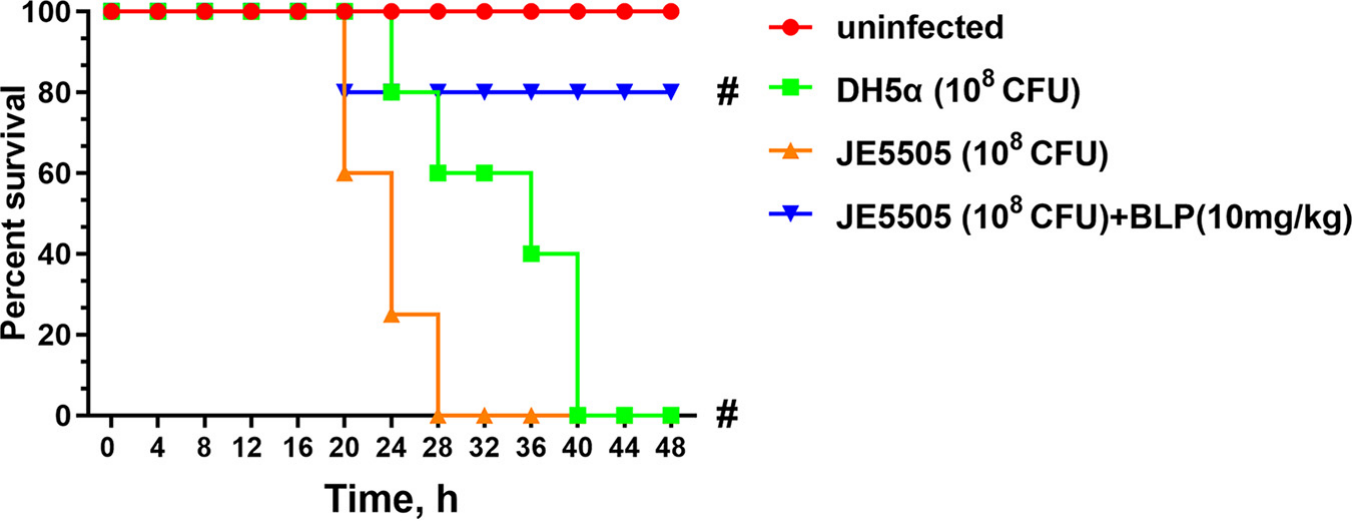
E. coli JE5505 has enhanced lethality compared with E. coli DH5α in mice. In each experimental group, mice (*n* = 20) were injected intraperitoneally with E. coli (1 × 10^8^ CFU; E. coli JE5505 was injected alone or in combination with 10 mg/kg BLP) or PBS (1 mL; uninfected). Differences in survival between the experimental groups were compared by the log rank test. ^#^, *P < *0.05, versus uninfected group.

As the effect of the presence of BLP on E. coli-induced mouse mortality is unknown, mouse mortality after E. coli DH5α and E. coli JE5505 injection (1 × 10^8^ colony forming units [CFU]) was analyzed. Mice injected intraperitoneally with E. coli DH5α for 28 h had a 60% survival rate compared with the uninfected control mice; however, mice injected intraperitoneally with E. coli JE5505 for 28 h had died. The survival rate of mice injected intraperitoneally with E. coli JE5505 accompanied by BLP (10 mg/kg of body weight) remained at 80% at 20 h postinfection. The E. coli DH5α-injected mice died at 40 h postinfection (Fig. 1). Such findings indicate that the bacterial outer membrane component BLP is essential for alleviating mortality in E. coli-infected mice.

### BLP is involved in the production of the proinflammatory cytokines, anti-inflammatory cytokines, and chemokines in E. coli-infected mice.

**FIG 2:**
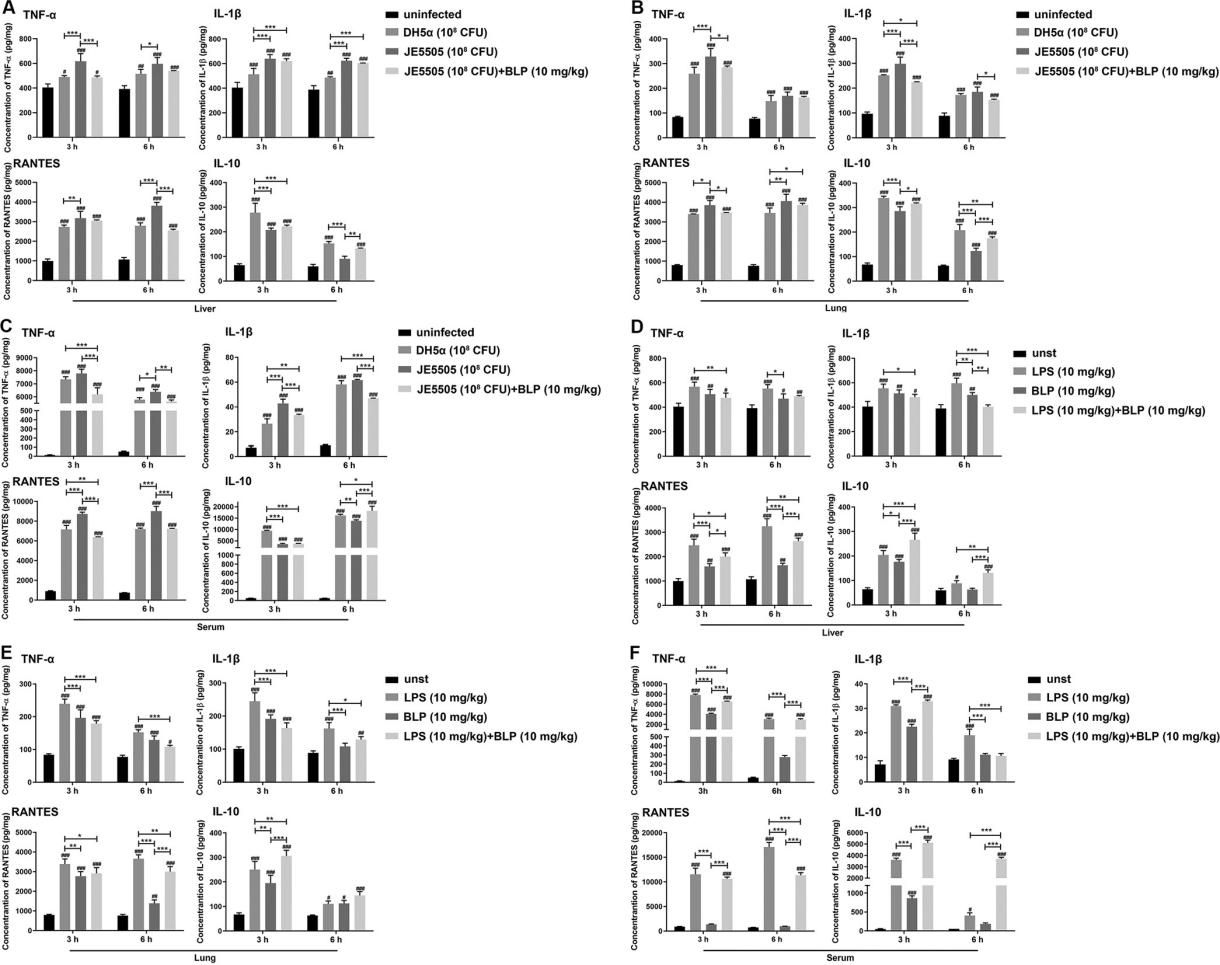
BLP is involved in proinflammatory cytokine, anti-inflammatory cytokine, and chemokine production in E. coli-infected mice. (A, B, C) Mice were injected intraperitoneally with E. coli (1 × 10^8^ CFU; E. coli JE5505 was injected alone or in combination with 10 mg/kg BLP) or PBS (1 mL; uninfected). (D, E, F) Mice were injected intraperitoneally with LPS (10 mg/kg) and BLP (10 mg/kg) alone or in combination or with PBS (1 mL; unst, unstimulated). The concentrations of TNF-α, IL-1β, RANTES, and IL-10 in the livers, lungs, and sera of mice were analyzed using ELISA (3 or 6 h after infection or stimulation). Results are expressed as mean ± SD of three independent experiments and were analyzed using two-way ANOVA with Bonferroni’s *post hoc* test. ^#^, *P < *0.05; ^##^, *P < *0.01; and ^###^, *P < *0.001 comparisons were to the respective control group. *, *P < *0.05; **, *P < *0.01; and ***, *P < *0.001 indicated statistically significant differences between two experimental groups.

Cytokines and chemokines are biomarkers of the host inflammatory response to bacterial infection. Here, we determined whether BLP is involved in the regulation of proinflammatory cytokines (TNF-α and IL-1β), anti-inflammatory cytokines (IL-10), and chemokine (RANTES) production in E. coli-infected mice (1 × 10^8^ CFU). Compared with those produced by E. coli DH5α-injected mice, E. coli JE5505-injected mice produced high levels of TNF-α, IL-1β, and RANTES and a low level of IL-10 in their livers, lungs, and sera after infection at indicated time points (3 h or 6 h postinfection; *P < *0.05) (Fig. 2A to C). Moreover, compared with those produced by E. coli JE5505-injected mice, E. coli JE5505 in combination with BLP-treated mice produced low levels of TNF-α, IL-1β, and RANTES in their livers, lungs, or sera at indicated time points (3 h or 6 h postinfection; *P < *0.05) (Fig. 2A to C). Furthermore, E. coli JE5505 in combination with BLP (10 mg/kg)-treated mice produced a high level of IL-10 in their livers, lungs, and sera at indicated time points (3 h or 6 h postinfection) compared with that produced by E. coli JE5505-injected mice (*P < *0.01) (Fig. 2A to C).

To investigate the roles of the two E. coli outer membrane components in the induction of cytokine and chemokine production in mice, mice were injected intraperitoneally with LPS (10 mg/kg) and BLP (10 mg/kg) alone or in combination. Both LPS and BLP induced TNF-α, IL-1β, RANTES, or IL-10 production in the livers, lungs, and sera of stimulated mice compared with those of the unstimulated controls (3 h or 6 h poststimulation; *P < *0.05) (Fig. 2D to F). Furthermore, compared with those produced by mice stimulated with LPS alone, LPS and BLP in combination induced low levels of TNF-α, IL-1β, and RANTES and a high level of IL-10 in the livers, lungs, and sera of mice after stimulation (3 h or 6 h poststimulation; *P < *0.05) (Fig. 2D to F). These findings align with those obtained in the E. coli-infected groups (Fig. 2A to C). Altogether, these findings suggest that E. coli- or LPS-induced proinflammatory cytokine and chemokine production could be attenuated and anti-inflammatory cytokine production could be enhanced by the presence of BLP.

### BLP could attenuate E. coli-induced HABP2 and HMGB1 expression in mice.

**FIG 3:**
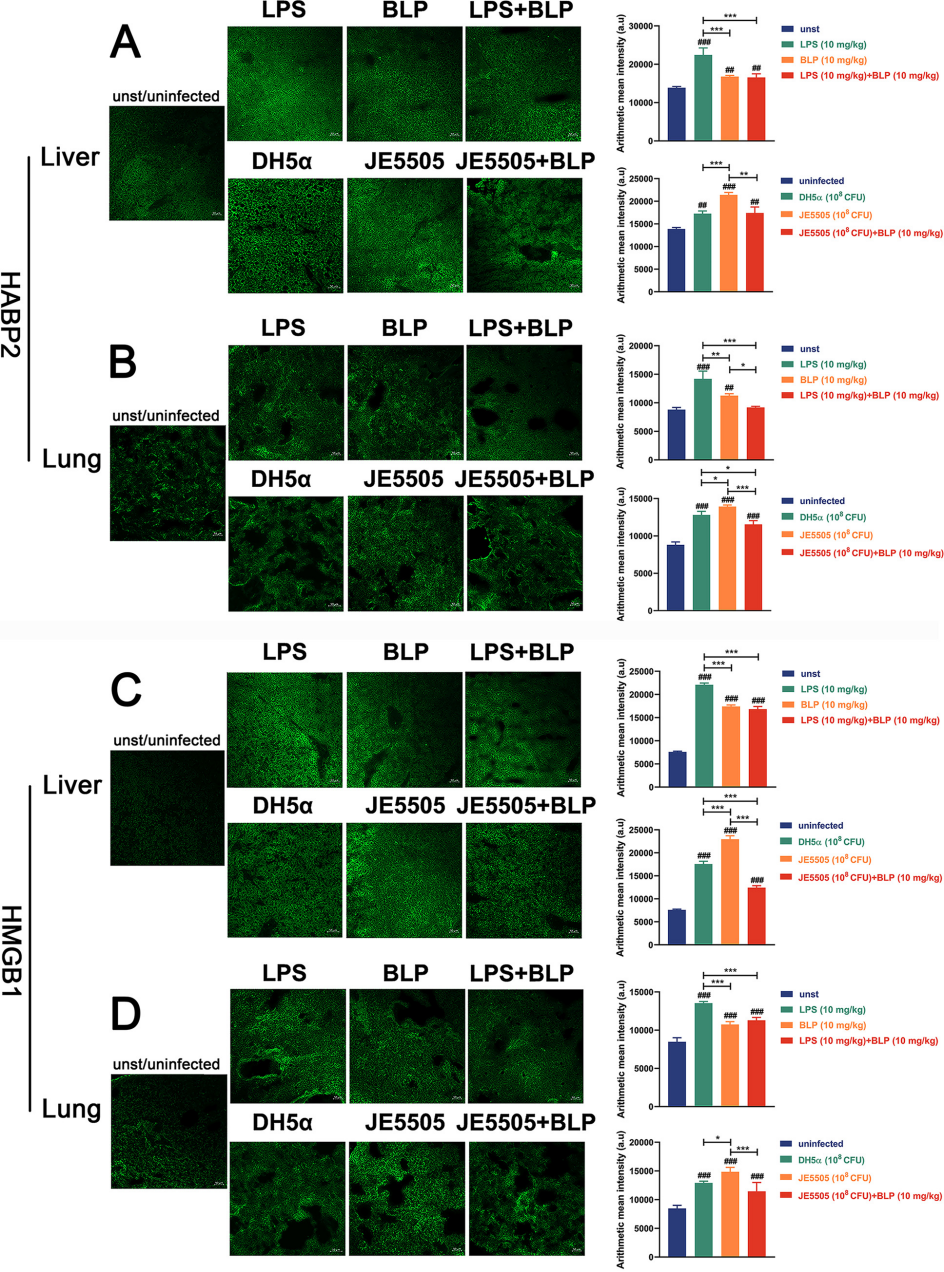
BLP could attenuate E. coli-induced HABP2 and HMGB1 expression in mice. (A to D) Mice were injected intraperitoneally with LPS (10 mg/kg) and BLP (10 mg/kg) alone or in combination, E. coli (1 × 10^8^ CFU; E. coli JE5505 was injected alone or in combination with 10 mg/kg BLP), or PBS (1 mL; unst, unstimulated). The protein expression level of HABP2 and HMGB1 (green) in the livers and lungs was determined with microscopy (12 h after stimulation or infection, ×100 magnification). The arithmetic mean intensities of HABP2 and HMGB1 expression were analyzed by Zen software (Zeiss; a. u, arbitrary unit). Results are expressed as mean ± SD of three independent experiments and were analyzed using two-way ANOVA with Bonferroni’s *post hoc* test. ^#^, *P < *0.05; ^##^, *P < *0.01; and ^###^, *P < *0.001 are shown as compared with their respective control group. *, *P < *0.05; **, *P < *0.01; and ***, *P < *0.001 indicated statistically significant differences between two experimental groups.

Hyaluronic acid binding protein 2 (HABP2) is associated with a variety of disease processes, including atherosclerosis, deep venous thrombosis, and inflammation (23, 24). High-mobility group box protein 1 (HMGB1), a signal of tissue damage, recruits mononuclear cells to sites of tissue damage to protect against possible infection (25). Here, mice were intraperitoneally injected with LPS (10 mg/kg) and BLP (10 mg/kg) alone or in combination, E. coli DH5α (1 × 10^8^ CFU), or E. coli JE5505 (1 × 10^8^ CFU, alone or in combination with BLP, 10 mg/kg) to determine whether BLP plays crucial roles in the regulation of organ damage. The expression levels of HABP2 and HMGB1 were assessed using fluorescence intensity through immunofluorescence assays. Compared with those of mice injected intraperitoneally with LPS, the expression levels of HABP2 and HMGB1 in the livers and lungs of mice stimulated with LPS in combination with BLP were decreased (*P < *0.001) (Fig. 3A to D). Furthermore, E. coli JE5505 induced high levels of HABP2 and HMGB1 expression in mouse livers or lungs compared with E. coli DH5α (*P < *0.05) (Fig. 3A to D). Moreover, E. coli JE5505-induced HABP2 and HMGB1 expression in livers and lungs were reduced by BLP treatment (*P < *0.01) (Fig. 3A to D). These findings suggest that BLP could attenuate liver and lung damage in mice following E. coli infection.

### The presence of BLP affects the activation of the signal transducer and activator of transcription 3 (STAT3), MAPK, and NF-κB signaling pathways in macrophages.

**FIG 4:**
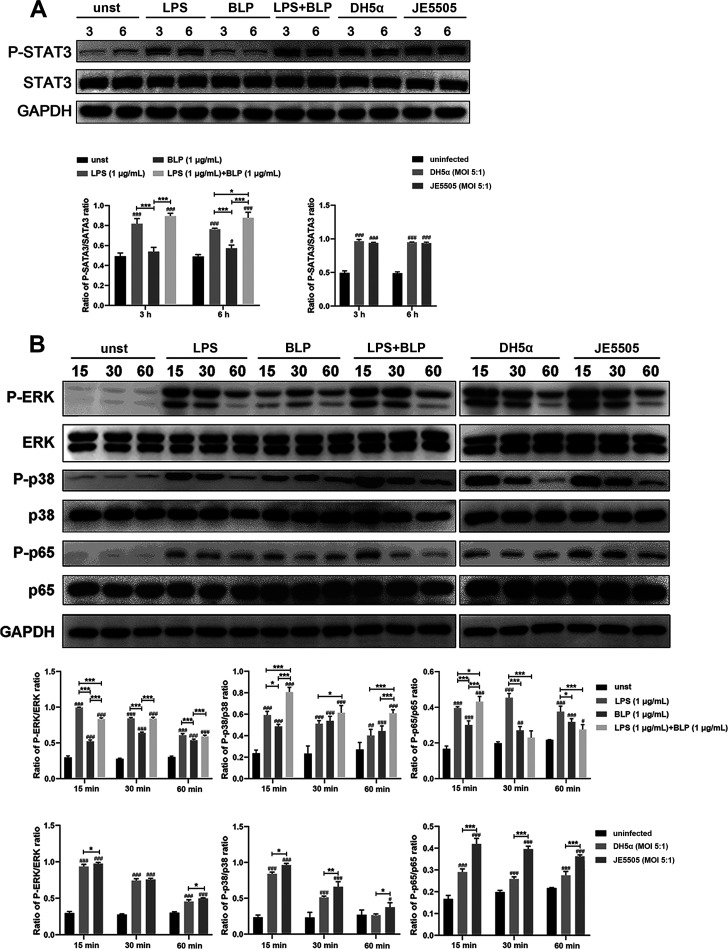
The presence of BLP affects the activation of the STAT3, MAPK, and NF-κB signaling pathways in macrophages. Macrophages were stimulated with LPS (1 μg/mL) and BLP (1 μg/mL) alone or in combination or stimulated with E. coli (MOI, 5:1) or left unstimulated (unst). (A) Activation of the STAT3 (P-STAT3) pathway was evaluated by Western blotting at 3 or 6 h poststimulation or infection. (B) Activation of the MAPK (P-ERK and P-p38) and NF-κB (P-p65) pathways was evaluated by Western blotting at 15 min, 30 min, and 60 min poststimulation or infection. GAPDH served as a loading control. Grayscale values were measured using ImageJ software. Results are expressed as mean ± SD of three independent experiments and were analyzed using two-way ANOVA with Bonferroni’s *post hoc* test. ^#^, *P < *0.05; ^##^, *P < *0.01; and ^###^, *P < *0.001 were groups compared with their respective control group. *, *P < *0.05; **, *P < *0.01; and ***, *P < *0.001 indicated statistically significant differences between two experimental groups.

STAT3 is the dominant mediator of the anti-inflammatory effects of IL-10 for the suppression of inflammation (26, 27). The activation of the MAPK and NF-κB pathways plays critical roles in inducing the secretion of numerous cytokines and chemokines (28–30). Thus, the roles of BLP in the activation of the STAT3, MAPK, and NF-κB signaling pathways in macrophages after stimulation with LPS (1 μg/mL) and BLP (1 μg/mL) alone or in combination, E. coli DH5α (MOI 5:1), or E. coli JE5505 (multiplicity of infection [MOI], 5:1) were examined by Western blotting. LPS and BLP in combination induced high levels of STAT3 phosphorylation at 6 h poststimulation compared with stimulation with LPS or BLP alone (*P < *0.05) (Fig. 4A). In the MAPK and NF-κB pathways, compared with macrophages stimulated with LPS alone, LPS and BLP in combination induced low levels of ERK (15 min poststimulation; *P < *0.001) and p65 (30 min and 60 min poststimulation; *P < *0.001) activation but enhanced p38 (15 min, 30 min, and 60 min poststimulation; *P < *0.05) and p65 (15 min poststimulation; *P < *0.05) activation (Fig. 4B). Meanwhile, E. coli JE5505 induced high levels of ERK, p38, and p65 activation at 15 min, 30 min, or 60 min postinfection compared with E. coli DH5α (Fig. 4B). These findings indicate that BLP could affect LPS- or E. coli-induced STAT3, MAPK, and NF-κB signaling activation in macrophages.

### E. coli-, LPS-, or BLP-induced cytokine and chemokine secretion in macrophages are mediated by the activation of MAPK and NF-κB signaling.

**FIG 5:**
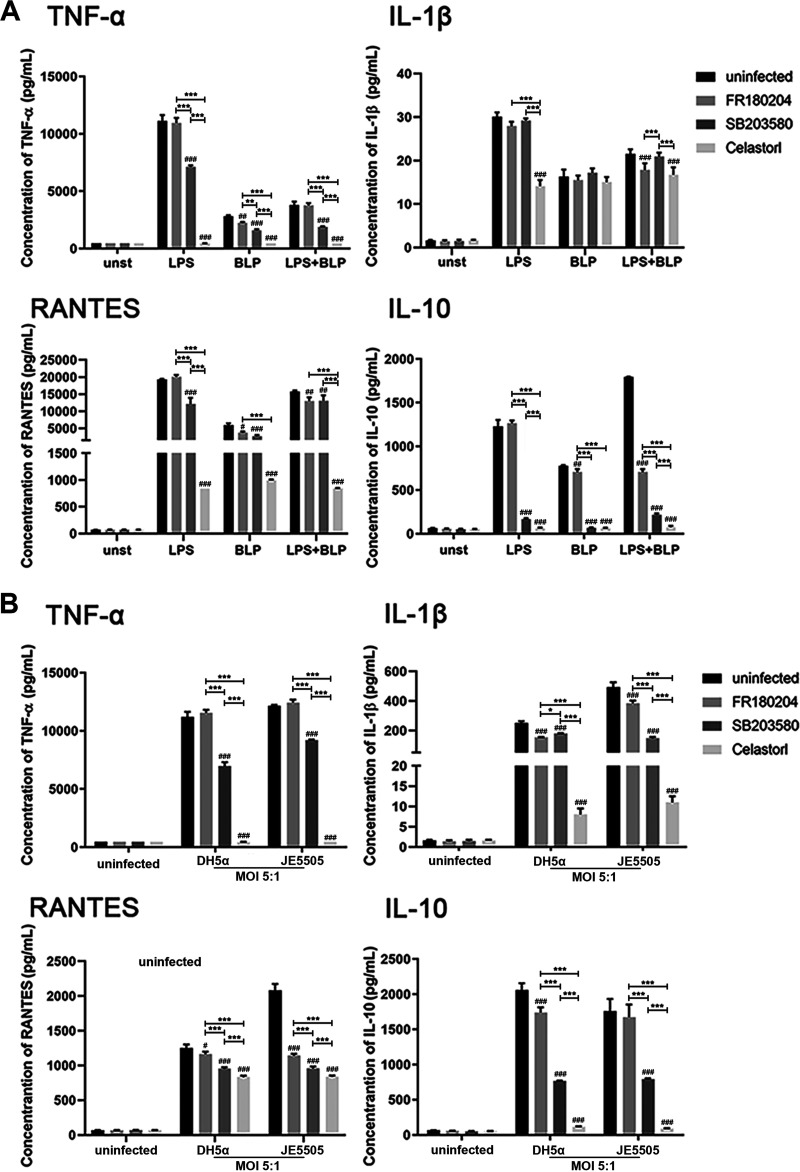
E. coli-, LPS-, or BLP-induced cytokine and chemokine secretion in macrophages are mediated by MAPK and NF-κB signaling activation. Macrophages were pretreated with the ERK inhibitor FR180204 (1 μM) or p38 inhibitor SB203580 (3 μM) before stimulation or treated with the NF-κB inhibitor celastrol (1 μM) in combination with stimulation. (A) Macrophages were stimulated with LPS (1 μg/mL) and BLP (1 μg/mL) alone or in combination or left unstimulated (unst). (B) Macrophages were infected with E. coli (MOI, 5:1) or left uninfected. Release of TNF-α, IL-1β, RANTES, and IL-10 into the supernatant of macrophage cultures was analyzed by ELISA, at 24 h after stimulation or infection. Results are expressed as mean ± SD of three independent experiments and were analyzed using two-way ANOVA with Bonferroni’s *post hoc* test. ^#^, *P < *0.05; ^##^, *P < *0.01; and ^###^, *P < *0.001 were groups compared with their respective control group. *, *P < *0.05; **, *P < *0.01; and ***, *P < *0.001 indicated statistically significant differences between two experimental groups.

Macrophages were pretreated with the ERK inhibitor FR180204 (1 μM), p38 inhibitor SB203580 (3 μM), or NF-κB inhibitor celastrol (1 μM) to further evaluate the roles of the MAPK and NF-κB pathways in E. coli*-*, LPS-, or BLP-induced cytokine release (24 h poststimulation or infection). FR180204 was found to partially inhibit BLP-induced (1 μg/mL) (alone or in combination with LPS [1 μg/mL]) cytokine and chemokine secretion but had no effect on cytokine and chemokine secretion in macrophages stimulated with LPS alone (*P < *0.05) (Fig. 5A). TNF-α, RANTES, and IL-10 secretion in macrophages stimulated with LPS and BLP alone or in combination were partially decreased by SB203580 treatment (*P < *0.01). However, SB203580 had no influence on IL-1β secretion in macrophages after stimulation (Fig. 5A). Cytokine and chemokine secretion in macrophages after stimulation with LPS alone or in combination with BLP were significantly decreased by celastrol treatment (*P < *0.001) but had no influence on IL-1β secretion in macrophages after stimulation with BLP alone (Fig. 5A). Furthermore, cytokine or chemokine secretion in macrophages after E. coli DH5α (MOI 5:1) and E. coli JE5505 stimulation (MOI 5:1) were decreased by FR180204, SB203580, or celastrol treatment (*P < *0.05) (Fig. 5B). These findings suggest MAPK and NF-κB signaling activation mediate bacterial component- or E. coli-induced cytokine and chemokine secretion in macrophages, and these processes might be more dependent on the activation of NF-κB signaling.

### The high levels of IL-10 production induced by BLP may not be the only motivating factor that alleviates mortality and organ damage in E. coli-infected mice.

**FIG 6:**
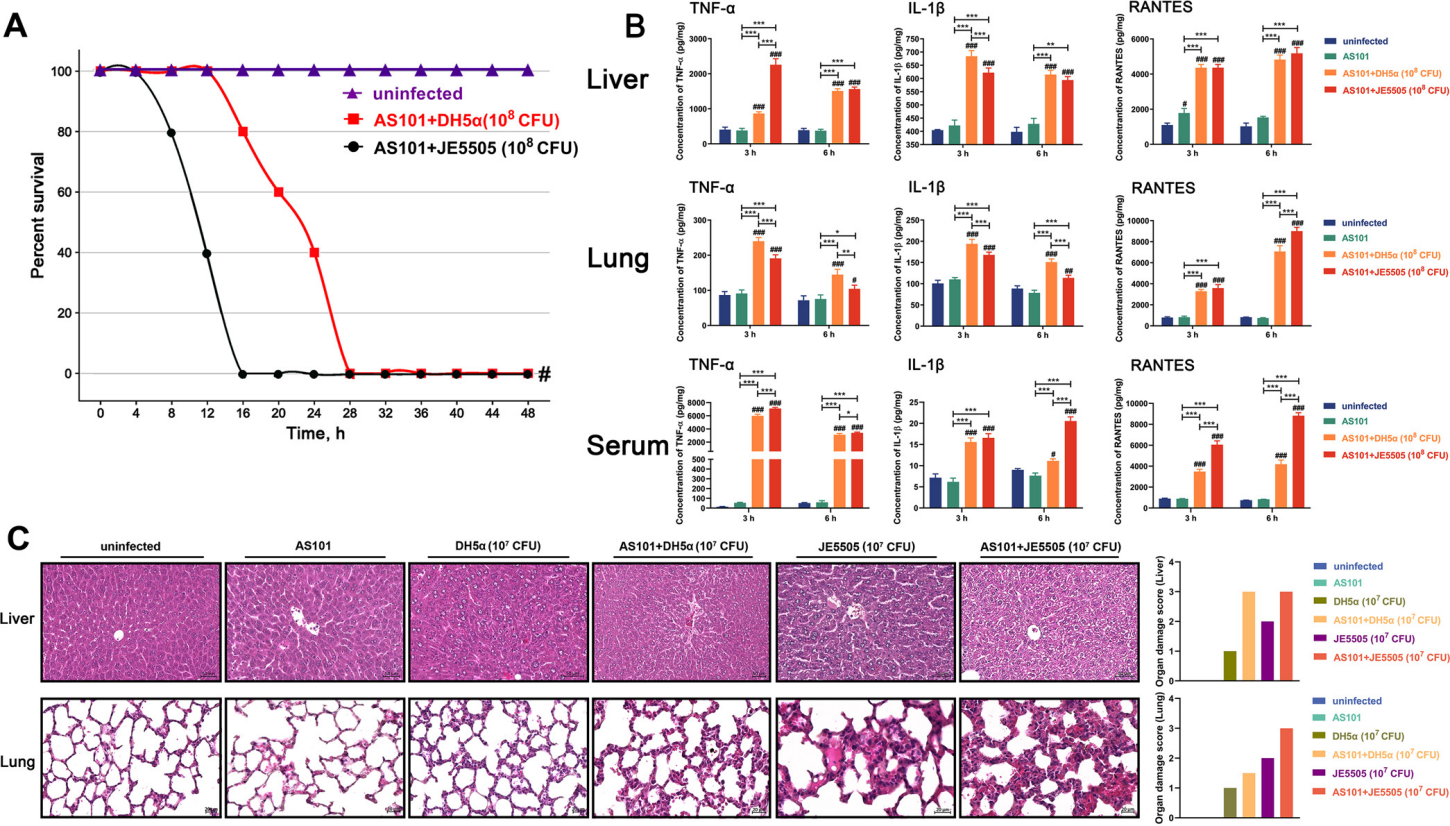
The high levels of IL-10 production induced by BLP may not be the only motivating factor that alleviates mortality and organ damage in E. coli-infected mice. Mice were pretreated with AS101 before infection. (A) Mice (*n* = 20) were injected intraperitoneally with E. coli (1 × 10^8^ CFU) or PBS (1 mL; uninfected). Differences in survival between the experimental groups were compared by the log rank test. ^#^, *P < *0.05 versus uninfected group. (B) Mice were injected intraperitoneally with E. coli (1 × 10^8^ CFU) or PBS (1 mL; uninfected). The concentration of TNF-α, IL-1β, RANTES, and IL-10 in the livers, lungs, and serum of mice was analyzed using ELISA (3 or 6 h poststimulation). (C) Mice were injected intraperitoneally with E. coli (1 × 10^7^ CFU) or PBS (1 mL, uninfected), and tissue sections were stained with H&E to reveal tissue damage (72 h postinfection). Results are expressed as mean ± SD of three independent experiments and were analyzed using two-way ANOVA with Bonferroni’s *post hoc* test. ^#^, *P < *0.05; ^##^, *P < *0.01; and ^###^, *P < *0.001 were compared with their respective control group. *, *P < *0.05; **, *P < *0.01; and ***, *P < *0.001 indicated statistically significant differences between two experimental groups.

The nontoxic immunomodulator AS101 is attributed to the direct inhibition of anti-inflammatory cytokine (IL-10) production (31). To further evaluate the protective roles of BLP against E. coli infection, AS101 was used to block the production of IL-10 in mice after infection (1 × 10^8^ CFU, shown in Fig. S1 in the supplemental material). Compared with mice that were not pretreated with AS101 (Fig. 1), an accelerated mortality caused by E. coli JE5505 or E. coli DH5α injection was observed in mice after the blockage of IL-10 production by AS101. Meanwhile, E. coli JE5505 still induced an accelerated mortality in mice compared with E. coli DH5α (Fig. 6A). A subsequent analysis revealed that compared with mice infected with E. coli DH5α, enhanced proinflammatory cytokine (TNF-α and IL-1β) and chemokine (RANTES) production were commonly observed in the sera of mice injected with E. coli JE5505 (3 or 6 h postinfection; *P < *0.05) (Fig. 6B). However, compared with E. coli DH5α-infected mice, we found that only TNF-α production in the livers at 3 h postinjection and RANTES production in the lungs at 6 h postinjection were enhanced in E. coli JE5505-injected mice (*P < *0.001) (Fig. 6B).

Liver and lung damage in mice exposed to E. coli (1 × 10^7^ CFU, with or without AS101 pretreatment, 72 h postinfection) were measured via morphological observations. Compared with mice that were not pretreated with AS101, E. coli JE5505 or E. coli DH5α was found to induce higher levels of liver and lung damage in those pretreated with AS101, which were characterized by diffuse vacuolar degeneration and widened interstitium with cellular infiltrations, respectively. Furthermore, E. coli JE5505 induced higher levels of liver or lung damage in mice than in E. coli DH5α, regardless of IL-10 production (Fig. 6C). These data demonstrate that, in addition to anti-inflammatory cytokine (IL-10) production, BLP could protect mice against E. coli-induced mortality and organ damage by attenuating proinflammatory cytokine and chemokine production.

## DISCUSSION

Excessive bacterial infection could lead to dysregulated systemic inflammatory response, and the common consequence of this occurrence is the development of sepsis or septic shock (32–34). Bacterial LPS is not the only endotoxin of Gram-negative bacteria, as the bacterial lipoproteins can trigger inflammatory responses similar to LPS (35). The bacterial outer membrane component BLP is involved in cytokine synthesis and endotoxin shock induced by E. coli (13, 16, 36). Interestingly, the bacterial lipoprotein could induce endotoxin-independent tolerance to septic shock and protects mice against LPS- and live bacterium-induced lethality (2). Tolerance is a critical component of the host response to infection, which could attenuate the host susceptibility to harm inflicted by pathogens or the immune system response (17, 18, 37). Previous studies have demonstrated that synthetic bacterial lipoprotein-induced tolerance could protect against bacterial lipoprotein-, LPS-, live bacterium-, and polybacterial sepsis-induced mortality (21, 22). The present study indicated that BLP plays a crucial role in protecting against E. coli-induced mortality and preventing excessive inflammatory responses in mice. Based on the result of a lethal experiment, it is speculated that there is a relationship between BLP and E. coli-induced mortality in mice (Fig. 1). Notable, Lakshmikanth et al. (13) reported that mice infected with DH5α died faster than those infected with JE5505 in Swiss albino mice, in contrast to the results in Fig. 1 and 6. The additional experiments using Swiss albino mice were performed (see Fig. S3 and Fig. S4 in the supplemental material). It was found that the survival rate of Swiss albino mice injected intraperitoneally with E. coli DH5α or E. coli JE5505 was 100% (72 h postinfection) (Fig. S3). In a previous study, both C57BL/6 and BALB/c mice were infected with E. coli, while C57BL/6 showed increased intestinal damage, altered integrity of the intestinal barrier, and impaired renal function that resulted in increased mortality (38). This phenomenon implies that it would be easier to observe BLP-induced tolerance in C57BL/6J mice than in other breeds.

Bacterial infection of cells activates inflammatory responses, which is the host protective response to ensure the removal of detrimental stimuli and the initiation of the healing process to repair damaged tissue (39). A key component of the inflammatory response in a host infected with bacteria is the upregulation of cytokine and chemokine secretion (30, 40). Proinflammatory cytokines, including TNF-α and IL-1β, mediate the inflammatory response at both the local and systemic levels, and E. coli induces large amounts of TNF-α expression *in vivo* (41–45). IL-10 plays essential roles in maintaining tissue homeostasis during infection and inflammation by restricting excessive inflammatory responses (46). Based on our findings, BLP could be contributed to attenuating the proinflammatory cytokine (TNF-α and IL-1β) and chemokine (RANTES) production and enhancing anti-inflammatory cytokine (IL-10) production in mouse sera, lungs, and livers after E. coli infection or LPS stimulation (Fig. 2). These results, particularly regarding cytokine and chemokine production in mice, are consistent with the mortality data generated in this study. Previous findings demonstrated that tolerance to synthetic bacterial lipoprotein may represent an essential regulatory mechanism during bacterial infection. For example, the bacterial lipoprotein can induce a cross-tolerance to LPS, which leads to decreased secretion of the proinflammatory cytokine TNF-α in human monocytic THP-1 cells (47). Moreover, a synthetic bacterial lipoprotein induces endotoxin-independent tolerance to septic shock and protects mice against LPS- and live bacterium-induced lethality (21). Based on the findings of previous studies and our results, we preliminarily conclude that BLP-induced tolerance could play protective roles in E. coli-infected mice by regulating inflammatory mediator production.

HABP2 and HMGB1 are considered signals of tissue damage associated with inflammatory response (25, 48). Consistent with the findings shown above, BLP contributes to attenuated HABP2 and HMGB1 expression in the livers and lungs of LPS-stimulated or E. coli-infected mice (Fig. 3). Such a finding provides further evidence that BLP could play protective roles in E. coli-infected mice. The outcome of infectious diseases is profoundly shaped by the interactions of microbes with host immunity (49). In general, high mortality and excess inflammation caused by infection do not mean bacterial success, as host death leads to the limitation of bacterial reproduction (50). Based on the findings of previous studies and our results, we infer that BLP-induced tolerance may be beneficial for E. coli by increasing bacterial reproduction through the suppression of host inflammatory response and host mortality.

In immune cells, the secretion of proinflammatory cytokines, chemokines, and anti-inflammatory cytokines (IL-10) can be induced by TLR-mediated MAPK and NF-κB signaling activation (30, 51, 52). Subsequently, the induction of STAT3 phosphorylation is required for the anti-inflammatory effects of IL-10, such as the suppression of proinflammatory cytokine production in macrophages and dendritic cells (53). Consistent with *in vivo* experimental data on mice, E. coli- or LPS-induced proinflammatory cytokine (TNF-α and IL-1β) and chemokine (RANTES) secretion were attenuated and anti-inflammatory cytokine (IL-10) secretion was enhanced by the presence of BLP in macrophages (shown in Fig. S2 in supplemental material). The presence of BLP could regulate the activation of MAPK and NF-κB signaling pathways and the secretion of inflammatory medium in LPS- and E. coli-stimulated macrophages (Fig. 4 and 5), indicating that the bacterial component- or E. coli-induced proinflammatory cytokine and chemokine secretion are likely to be primarily dependent on the activation of NF-κB (p65) signaling. In addition, the secretion of anti-inflammatory cytokines appears to be more closely associated with the activation of both MAPK (p38) and NF-κB (p65) signaling pathways. During stimulation, synthetic bacterial lipoprotein-induced tolerance is characterized by an attenuated proinflammatory cytokine production in the host (47). Even though the production of IL-10 was blocked in mice, the mortality caused by E. coli JE 5505 infection remained at high levels, as did the cytokines in their serum and the organ damage, compared with E. coli DH5α-infected mice (Fig. 6A to C). These results suggest that the protective effect of BLP-induced tolerance is not limited to the regulation of IL-10 production, and other mechanisms may also be involved in protecting mice from E. coli-induced mortality. Therefore, more research is needed to investigate the intrinsic tolerance mechanism.

In conclusion, the BLP component of the E. coli outer membrane plays a crucial role in modulating the immune response to E. coli infection. Our study indicates that BLP-induced tolerance could attenuate mortality and the severity of organ damage, which could be achieved by regulating the production of proinflammatory and anti-inflammatory cytokines with the involvement of the MAPK and NF-κB signaling pathways. These findings demonstrated that BLP-induced tolerance could be involved in E. coli infection-induced immune response and might provide a novel strategy to combat bacterial infection.

## MATERIALS AND METHODS

### Ethics statement.

All animal experiments were performed according to regulations of the Administration of Affairs Concerning Experimental Animals in China. The experimental protocol was approved by the Animal Welfare and Research Ethics Committee of the Inner Mongolia Agricultural University (approval identifier [ID] NND2021013).

### Bacterial strains and animals.

E. coli DH5α (BLP-positive, E. coli K-12 derivative) bacteria were stored in our laboratory. The E. coli JE5505 (BLP-negative, E. coli K-12 derivative) strain was obtained from The Coli Genetic Stock Center (CGSC) of Yale University (New Haven, CT; CGSC number 6672). Although the E. coli JE5505 strain lacks BLP, its normal physiological properties of growth and multiplication are not impaired (54). According to previously reported studies, E. coli DH5α was referenced for E. coli JE5505 during experimental infection (13, 55). To determine the number of CFU in the bacterial suspension used in this study, 1 mL of bacterial suspension (1 × 10^7^ CFU/mL) was seeded in 100 mL of Luria-Bertani (LB; Oxoid, Basingstoke, UK) broth and incubated with shaking at 37°C for 12 h (optical density at 600 nm [OD_600_] value of approximately 0.9 in the log phase). Thereafter, the cultured bacterial suspension was serially diluted and plated on LB agar plates and incubated at 37°C for 12 h. The colony counting technique was used to determine the total number of CFU (approximately 1 × 10^8^ CFU/mL). At least eight independent experiments were performed, and the results of CFU counting were consistent. C57BL/6J mice were provided by the Model Animal Research Center of Nanjing University, Nanjing, China.

### Free form of BLP purification.

E. coli DH5α was grown in LB Broth, pelleted, and stored frozen. We used Inouye’s procedure to purify the free form of murein lipoprotein from E. coli, as described previously (12, 13, 56). Briefly, bacteria were resuspended in 1 mL of S-buffer (10 mM sodium phosphate [pH 7.5] and 5 mM EDTA) with 1 mM phenylmethylsulfonyl fluoride (PMSF) per gram of wet bacteria and lysed on ice by sonication. After unlysed cells were removed via centrifugation (1,000 × *g* for 15 min at 4°C), the cell membranes were collected (40,000 × *g* for 40 min at 4°C) and resuspended in S-buffer. Thereafter, 4% SDS and 0.5% 2-mercaptoethanol (2-ME) was added to the cell suspension, which was boiled for 30 min, stirred overnight at 25°C, and then centrifuged (50,000 × *g* for 30 min at 23°C) to collect the supernatant. Contaminating proteins were precipitated at low pH using 5% acetone before the collection of BLP as a precipitate using 30% acetone. The precipitate was renatured using 1% SDS. To remove LPS from the BLP, BLP was extracted using phenol and then recovered from the phenol phase by precipitation using 30% acetone. We repeated the acetone fractionation several times, and after the third or fourth acetone fractionation, one major band was observed on a silver-stained polyacrylamide gel. The protein concentration was determined using the bicinchoninic acid (BCA) protein assay kit (Thermo Scientific, Rockford, IL). The LPS content of our purified BLP at the employed dilutions was less than 0.5 EU/g BLP, as determined by the limulus amebocyte lysate assay (QCL-1000; BioWhittaker, Walkersville, MD).

### Experimental infection and treatment *in vivo*.

In each experimental group, 8-week-old C57BL/6J mice were injected intraperitoneally with LPS from E. coli (10 mg/kg; ultrapure LPS-B5 underwent enzymatic treatment to remove lipopeptides; InvivoGen, San Diego, CA) and BLP (10 mg/kg) alone or in combination, E. coli DH5α (1 × 10^7^ CFU or 1 × 10^8^ CFU) or E. coli JE5505 (1 × 10^7^ CFU or 1 × 10^8^ CFU) alone or in combination with BLP. Serum was collected at 3 h and 6 h posttreatment, while the livers and lungs were harvested at 3, 6, 12, and 72 h posttreatment. In the groups treated with the nontoxic IL-10 inhibitor ammonium trichloro (dioxoethylene-o,o’) tellurate (AS101; Tocris, Bristol, UK), each C57BL/6J mouse was injected intraperitoneally with 10 μg AS101 for 24 h before infection. Thereafter, each mouse was injected intraperitoneally with 10 μg AS101 and E. coli (DH5α or JE5505, 1 × 10^7^ CFU or 1 × 10^8^ CFU) in combination, as described previously (57).

### Experimental infection and treatment *in vitro*.

Three days before the extraction of peritoneal macrophages, 8-week-old C57BL/6J mice were injected intraperitoneally with 2 mL of 3% thioglycolate medium (BD Biosciences, Sparks, MD). Peritoneal macrophages were isolated by washing the peritoneal cavity with endotoxin-free phosphate-buffered saline (PBS; HyClone, Logen, UT) and cultured at 37°C in 5% CO_2_ in RPMI 1640 media supplemented with 10% fetal bovine serum (HyClone). The cells were seeded in 6-well culture plates (2 × 10^6^/well) in 1 mL of fresh culture medium and washed thrice with PBS before stimulation. Macrophages were stimulated with LPS (1 μg/mL) and BLP (1 μg/mL) alone or in combination or stimulated with E. coli (DH5α or JE5505, 1 × 10^7^ CFU/well, multiplicity of infection [MOI] of 5:1). After 1 h of infection, extracellular E. coli was eliminated via washing with fresh medium containing 10 mg/mL ovalbumin and 20 U/mL interferon gamma (58). In the groups treated with the ERK inhibitor FR180204 (Tocris, Bristol, UK) or p38 inhibitor SB203580 (Tocris), macrophages were incubated in culture media supplemented with 1 μM FR180204 for 2 h or 3 μM SB203580 for 30 min before stimulation. In the groups treated with the NF-κB inhibitor celastrol (InvivoGen, San Diego, CA), macrophages were incubated in culture media supplemented with 1 μM celastrol in combination with stimulation.

### Enzyme-linked immunosorbent assay (ELISA).

The harvested livers and lungs were cut into pieces and weighed. Thereafter, the tissues were homogenized and lysed with the tissue protein extraction reagent (T-PER; Thermo Scientific, Waltham, MA) and centrifuged at 1,500 × *g* for 20 min at 4°C. After collection, the mouse sera were incubated at 37°C for 1 h and further incubated at 4°C overnight. Subsequently, the sera were centrifuged at 1,000 × *g* for 15 min at 25°C and the supernatants were collected for subsequent tests. The supernatants of macrophages cultured in 6-well plates were centrifuged at 300 × *g* for 8 min at 4°C and stored at −80°C. The concentration of cytokines and chemokines in the tissue extracts, serum, and cultured supernatants of macrophages were measured using mouse ELISA kits for TNF-α, IL-1β, IL-10 (Biolegend, San Diego, CA), and RANTES (PeproTech, Rocky Hill, NJ) according to the manufacturer’s instructions. The experiment was performed in triplicates.

### Western blot analysis.

For total cellular protein extraction, macrophages were treated with the mammalian protein extraction reagent (M-PER; Thermo Scientific, Waltham, MA). The concentrations of protein samples were determined using BCA assay kits (Thermo Scientific, Rockford, IL). For Western blot analysis, 10 μg of total protein per lane was resolved by sodium dodecyl sulfate-polyacrylamide gel electrophoresis and transferred onto polyvinylidene difluoride membranes. Membranes were blocked with StartingBlock (TBS) blocking buffer (Thermo Scientific, Rockford, IL) for 1 h at 25°C and then incubated with primary antibody for 14 h at 4°C. Rabbit anti-phospho-ERK, anti-ERK, anti-phospho-p38, anti-p38, anti-phospho-NF-κB p65, anti-NF-κB p65, anti-phospho-STAT3, and anti-STAT3 monoclonal antibodies (1:1,000, Cell Signaling Technology, Beverly, MA), along with rabbit anti-GAPDH (1:10,000, Abcam, Cambridge, UK) were used for protein detection. Proteins were visualized using secondary horseradish peroxidase (HRP)-conjugated goat anti-rabbit antibody (1:10,000, Cell Signaling Technology) and Pierce SuperSignal West Femto chemiluminescent substrate (Thermo Scientific, Rockford, IL). Grayscale values of bands generated by Western blotting were measured using ImageJ software (version 1.48v; National Institutes of Health, Bethesda, MD).

### Immunofluorescence assays and morphological observation of the livers and lungs.

Frozen 6-μm sections of treated liver and lung tissues from mice injected intraperitoneally with LPS and BLP (alone or in combination), E. coli DH5α, or E. coli JE5505 (alone or in combination with BLP) were thawed at room temperature for 15 min and fixed in cold acetone for 10 min. Thawed sections were washed using cold PBS containing 0.25% Tween and blocked for 1 h using 5% bovine serum albumin at 25°C. A rabbit anti-HMGB1 polyclonal antibody (1:200; Novus Biologicals, Littleton, CO) and anti-HABP2 monoclonal antibody (1:100; Abcam, Cambridge, UK) were added, and the sections were incubated overnight at 4°C in the dark. After incubation, the slides were washed thrice for 15 min each using PBS containing 0.25% Tween and incubated in an Alexa Fluor 488-conjugated goat anti-rabbit IgG (H&L) secondary antibody (1:1000, Abcam) for 1 h at room temperature in the dark. A confocal microscope (LSM 800; Zeiss, Oberkochen, Germany) was used to capture images (×100 magnification) to analyze fluorescence intensity. One of the three captured fields was randomly selected from each sample and used for fluorescence intensity analysis. Images from different samples were captured under identical conditions. Mouse liver and lung tissues were collected to prepare paraffin sections, which were dehydrated with various concentrations of alcohol (100%, 75%, 50%, and 25%). After hematoxylin and eosin (H&E) staining, pathological changes in the liver and lung tissues were observed under an Axio Scan.Z1 slide scanner (Zeiss, Thornwood, NY).

### Data analysis.

All data were analyzed using Prism 8 (GraphPad InStat Software, San Diego, CA) and are expressed as mean ± standard deviation (SD). Statistical significance was evaluated using two-way analysis of variance (ANOVA) with Bonferroni’s *post hoc* test. Statistical significance was defined as a *P* value of <0.05.
