# Supplementary material for: Braun Lipoprotein Protects against Escherichia coli-Induced Inflammatory Responses and Lethality in Mice
Source: Microbiol Spectr. 2023 Mar 14;11(2):e03541-22. doi: 10.1128/spectrum.03541-22 (PMC10100777; doi:10.1128/spectrum.03541-22)
Supplement: Supplemental file 1 — Fig. S1 to S4. Download spectrum.03541-22-s0001.pdf, PDF file, 0.8 MB [file spectrum.03541-22-s0001.pdf]

Supplementary Material

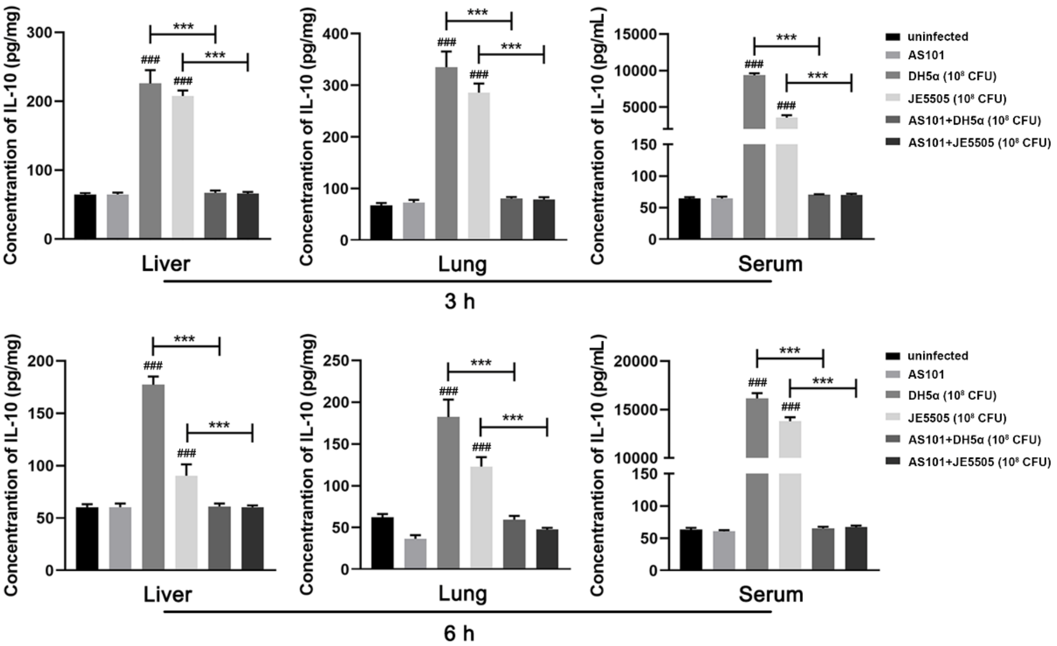

Supplemental Fig. S1. IL-10 production was inhibited by AS101 treatment in *E. coli*-injected mice. Mice were pretreated with AS101 before *E. coli* injection. Mice were injected intraperitoneally with *E. coli* (1×10^8 CFU) or PBS (1 mL, uninfected). The concentration of IL-10 in the livers, lungs, and serum of mice were analyzed using ELISA (3 or 6 h post-stimulation). Results are expressed as the mean ± SD of three independent experiments and were analyzed using one-way ANOVA followed by Tukey's post-hoc test. $^{\#}P < 0.05$, $^{\#\#}P < 0.01$, and $^{\#\#\#}P < 0.001$ compared to the uninfected group. $^{*}P < 0.05$, $^{**}P < 0.01$, and $^{***}P < 0.001$ indicated statistically significant differences between two experimental groups.

Supplementary Material

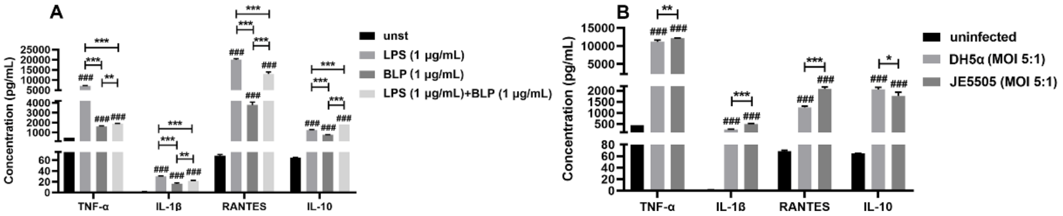

Supplemental Fig. S2. BLP is involved in proinflammatory cytokine, anti-inflammatory cytokine, and chemokine secretion in *E. coli*-infected mice. **(A)** Macrophages were stimulated with LPS (1 μg/mL) and BLP (1 μg/mL) alone or in combination, or left unstimulated (unst). **(B)** Macrophages were infected with *E. coli* (MOI 5:1) or left unstimulated. The release of TNF-α, IL-1β, RANTES, and IL-10 into the supernatant of macrophage cultures was analyzed using ELISA 24 h after stimulation or infection. Results are expressed as the mean ± SD of three independent experiments and were analyzed using two-way ANOVA with Bonferroni's post hoc test. $^{\#}P < 0.05$, $^{\#\#}P < 0.01$, and $^{\#\#\#}P < 0.001$ compared to the respective control group. $^{*}P < 0.05$, $^{**}P < 0.01$, and $^{***}P < 0.001$ indicated statistically significant differences between two experimental groups.

Supplementary Material

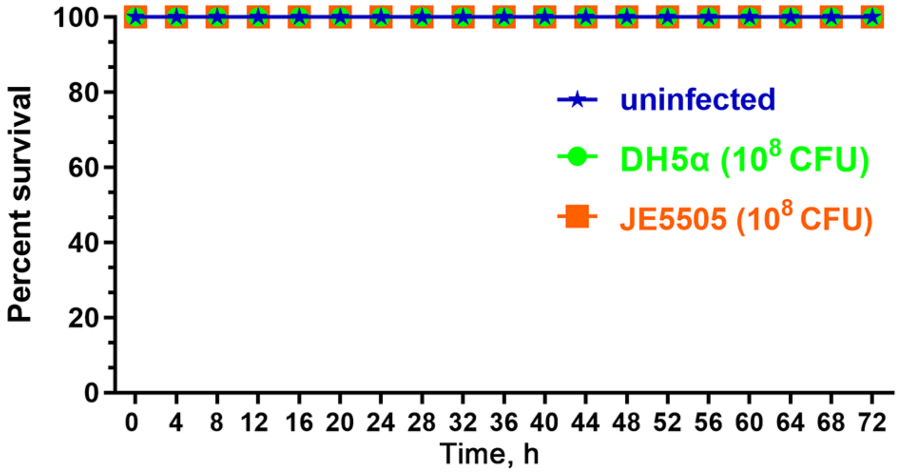

Supplemental Fig. S3. There was no significant effect on mortality in Swiss albino mice after intraperitoneal injection of *E. coli*. In each experimental group, Swiss albino mice (n = 20) were injected intraperitoneally with *E. coli* (1 × 10^8 CFU or PBS (1 mL, uninfected). Differences in survival between the experimental groups were compared by the log rank test. # $P < 0.05$, vs. uninfected group.

Supplementary Material

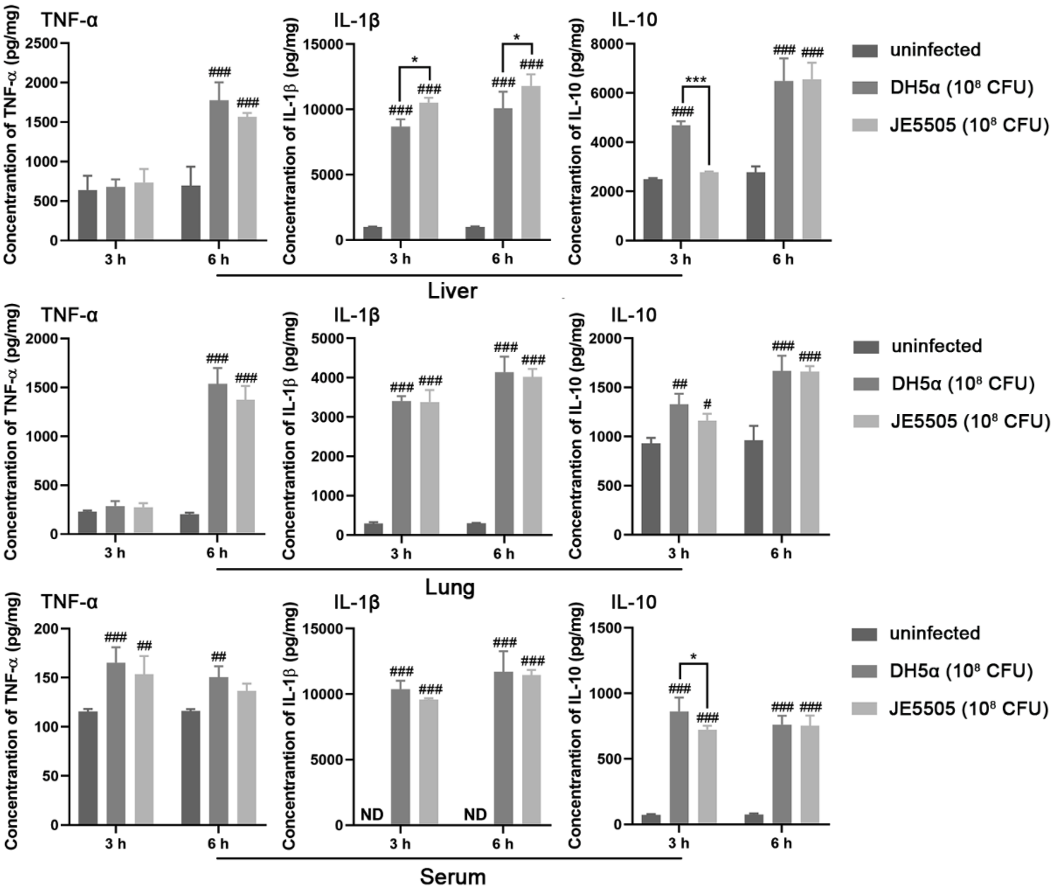

Supplemental Fig. S4. The effect of BLP on proinflammatory cytokine and anti-inflammatory cytokine in *E. coli*-infected Swiss albino mice. Swiss albino mice were injected intraperitoneally with *E. coli* (1 × 10^8 CFU or PBS (1 mL, uninfected). The concentration of TNF-α, IL-1β and IL-10 in the livers, lungs, and sera of mice was analyzed using ELISA (3 and 6 h after infection). Results are expressed as mean ± SD of three independent experiments and were analyzed using two-way ANOVA with Bonferroni's post-hoc test. #$P < 0.05$, ##$P < 0.01$, and ###$P < 0.001$ compared to the respective control group. *$P < 0.05$, **$P < 0.01$, and ***$P < 0.001$ indicated statistically significant differences between two experimental groups.
